# Oxidized Forms of Ergothioneine Are Substrates for Mammalian Thioredoxin Reductase

**DOI:** 10.3390/antiox11020185

**Published:** 2022-01-19

**Authors:** Kaelyn A. Jenny, Gracyn Mose, Daniel J. Haupt, Robert J. Hondal

**Affiliations:** 1 Room B413, Given Laboratory, Department of Biochemistry, College of Medicine, University of Vermont, 89 Beaumont Ave, Burlington, VT 05405, USA; Kaelyn.Jenny@uvm.edu (K.A.J.); gracynmose@gmail.com (G.M.); 2 Room E340, Innovation Hall, Department of Chemistry, University of Vermont, 82 University Place, Burlington, VT 05405, USA; haupt@beamtx.com

**Keywords:** ascorbate, ergothioneine, thioredoxin reductase, glutathione reductase, selenium, oxidative stress, antioxidant, selenoneine, 2-thiohistidine

## Abstract

Ergothioneine (EGT) is a sulfur-containing amino acid analog that is biosynthesized in fungi and bacteria, accumulated in plants, and ingested by humans where it is concentrated in tissues under oxidative stress. While the physiological function of EGT is not yet fully understood, EGT is a potent antioxidant in vitro. Here we report that oxidized forms of EGT, EGT-disulfide (ESSE) and 5-oxo-EGT, can be reduced by the selenoenzyme mammalian thioredoxin reductase (Sec-TrxR). ESSE and 5-oxo-EGT are formed upon reaction with biologically relevant reactive oxygen species. We found that glutathione reductase (GR) can reduce ESSE, but only with the aid of glutathione (GSH). The reduction of ESSE by TrxR was found to be selenium dependent, with non-selenium-containing TrxR enzymes having little or no ability to reduce ESSE. In comparing the reduction of ESSE by Sec-TrxR in the presence of thioredoxin to that of GR/GSH, we find that the glutathione system is 10-fold more efficient, but Sec-TrxR has the advantage of being able to reduce both ESSE and 5-oxo-EGT directly. This represents the first discovered direct enzymatic recycling system for oxidized forms of EGT. Based on our in vitro results, the thioredoxin system may be important for EGT redox biology and requires further in vivo investigation.

## 1. Introduction

Ergothioneine (EGT) is a redox-active sulfur-containing amino acid derivative that is biosynthesized from the coupling of histidine (His) and cysteine (Cys) in fungi, actinobacteria, and cyanobacteria [1,2,3,4,5,6,7,8,9,10,11]. It is the betaine (quaternary ammonium group) of 2-thiohistidine (2-thioHis). Plants do not synthesize EGT but accumulate it through symbiotic relationships with fungi and bacteria [1,2,3,4,5,6,7,8,9,10]. EGT is subsequently ingested and concentrated in animal tissue through a cation/carnitine transporter protein known as OCTN1, especially in tissue that is susceptible to oxidative stress [4,5,6,7,8,9,10,11,12]. A related compound to EGT is selenoneine, in which selenium replaces the sulfur atom of EGT [13,14].

While the exact role of EGT in vivo has not been determined, it possesses remarkable antioxidant and cytoprotective properties in vitro. For example, EGT has been shown to be a powerful scavenger of singlet oxygen (^1^O_2_), hydroxyl radicals, and hypochlorite (HOCl) in vitro with the ability to protect other molecules from oxidative damage [2,3,4,5,6,7,8,9,15,16,17,18,19,20]. Experiments have shown that OCTN1 knockout cells are more prone to DNA damage, protein oxidation, and lipid peroxidation, suggesting EGT functions as an antioxidant and cytoprotective agent in vivo [5]. Another possible function for EGT is in the detoxification of xenobiotic electrophiles [21].

Dietary EGT has recently been shown to be an independent marker for lowered risk of cardiovascular disease in humans [22]. Similarly, lower EGT levels in the blood have been shown to be correlated with frailty in elderly populations [23]. Because it is a dietary-derived organic molecule that has been shown to have some health benefits, it has been referred to as a “potential vitamin” or “candidate vitamin” [5,24,25].

**Figure 1:**
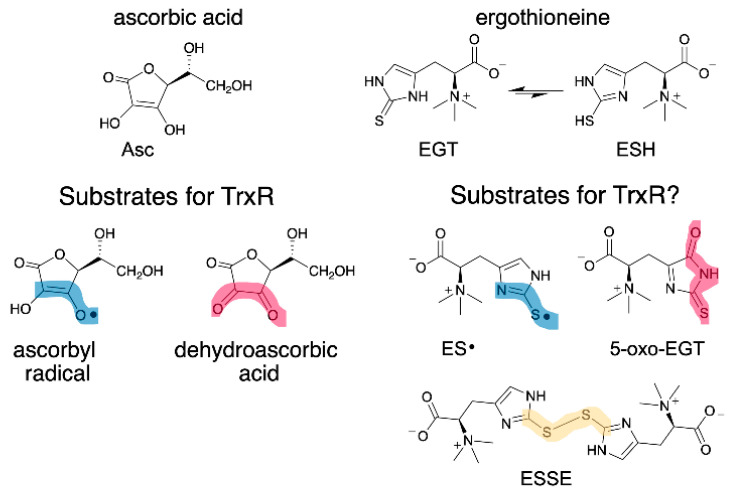
Structure of EGT, Asc and their oxidized forms. (**Left**) Asc• and dehydroascorbate are known substrates for Sec-TrxR [26,27]. (**Right**) EGT has two tautomeric forms: thione (EGT) and thiol (ESH), with the thione being highly favored in neutral aqueous solution. This study investigated whether oxidized forms of EGT are also substrates for Sec-TrxR. The oxidized forms of EGT and Asc have clear structural similarities, as highlighted in blue and red. Sec-TrxR, a selenoenzyme, is also able to reduce the number of other small molecule substrates, including *S*-nitrosoglutathione, lipoic acid/lipoamide, lipid hydroperoxides, and ubiquinone [28,29,30,31].

EGT has a similar function as vitamin C (ascorbate, Asc), as both are antioxidants that can donate H• to quench radicals. As such, we wondered whether oxidized forms of EGT could be reduced by mammalian thioredoxin reductase (Sec-TrxR) since Sec-TrxR recycles oxidized forms of Asc, ascorbyl radical (Asc•) and dehydroascorbate, back to the reduced form [26,27]. Oxidized forms of EGT include the EGT radical (ES•), the disulfide of EGT (ESSE), and (2*S*)-3-(5-oxo-2-thioxoimidazoline-4-yl)-2-(trimethylazaniumyl)propanoate (abbreviated hereafter as 5-oxo-EGT) [15,20]. These oxidized products of EGT (Figure 1) result from a reaction with hydroxyl radical (ES•), various oxidants including hydrogen peroxide (ESSE), and singlet oxygen (5-oxo-EGT).

In this report, we tested whether Sec-TrxR or glutathione reductase (GR) could reduce the oxidized forms of EGT shown in Figure 1. TrxR is part of the thioredoxin system comprised of TrxR, thioredoxin (Trx), and NADPH [32,33,34]. GR is part of the glutathione system, which is made up in part by GR, glutathione (GSH), and NADPH [35].

We found that both GR and Sec-TrxR could reduce the oxidized forms of EGT, but GR could not catalyze the reduction in the oxidized forms without the addition of GSH to the assay. In contrast, selenium-containing Sec-TrxR was able to efficiently reduce all of the oxidized forms tested without the addition of an exogenous thiol. Our in vitro results imply that the thioredoxin system may make an important contribution to the redox biology of EGT in mammalian systems.

## 2. Materials and Methods

### 2.1. Materials

Cys free-base was purchased from Calbiochem (Billerica, MA, USA). Histidine·HCl monohydrate and 3-mercaptopropionic acid were purchased from ACROS Organics (Pittsburgh, PA, USA). Rose bengal and bromine were purchased from Sigma-Aldrich (Milwaukee, WI, USA). Deuterium oxide (D, 99.9%) was purchased from Cambridge Isotope Laboratories (Andover, MA, USA). Hydrogen peroxide (30% in water) was purchased from Fisher Scientific (Waltham, MA, USA). All other chemicals were purchased from Sigma-Aldrich, Fisher Scientific, or ACROS Organics. ^1^H-NMR and ^13^C-NMR spectra were recorded with a Bruker Advance III HD 500 MHz NMR spectrometer (Bruker, Billerica, MA, USA). All enzyme kinetics assays were performed on a Cary 50 ultraviolet−visible (UV−Vis) spectrophotometer (Varian, Walnut Creek, CA, USA). Mass spectrometric (MS) analysis was either performed on a Thermo Scientific™ Q Exactive™ Hybrid Quadrupole-Orbitrap Mass Spectrometer (Waltham, MA, USA) or an Applied Biosystems QTrap 4000 hybrid triple-quadrupole/linear ion trap liquid chromatograph-mass spectrometer (LCMS) (SciEx, Framingham, MA, USA). The visible light source was a 500 W WorkForce™ lamp with a UV filter. Ergothioneine was a gift from Dr. E. Will Taylor of the University of North Carolina-Greensboro. Selenoneine was a gift from Dr. Florian Seebeck of the University of Basel.

### 2.2. Synthesis of L-2-Thiohistidine

L-2-thiohistidine was synthesized according to the procedure from Erdelmeier et al., 2012 [36]. This reaction works best when performed on a 10 g or higher scale. Histidine·HCl monohydrate (14 g, 66.8 mmol, 1.0 eq.) was dissolved in 134 mL of deionized water. After the His was fully dissolved, the solution was cooled in an ice bath to 0 °C. Once the reaction was cooled, bromine (4.45 mL, 86.8 mmol, 1.3 eq.) was added rapidly dropwise, resulting in a bright orange solution. After 6 min, free-base Cys (24.3 g, 200.4 mmol, 3.0 eq.) was added to the reaction, resulting in a yellow solution. The solution was stirred at 0 °C for 1 h. An oil bath was preheated to 95 °C. After 1 h, 3-mercaptopropionic acid (34.9 mL, 400.7 mmol, 6.0 eq.) was added to the reaction, and the reaction was transferred to the oil bath at 95 °C. A condenser was attached to the reaction, and the reaction was stirred for 18 h at 95 °C, after which the reaction had turned dark brown. The reaction was removed from the oil bath and condenser and allowed to cool to room temperature. The aqueous solution was then extracted with ethyl acetate (4×, 100 mL). The aqueous layer remained dark brown after extraction. The aqueous layer was transferred to a clean flask and placed in an oil bath preheated to 40 °C. Nitrogen gas was bubbled through the solution at 40 °C to help remove excess HBr in solution. The pH of the solution was adjusted to 6.5 with 30% ammonia hydroxide while nitrogen was being bubbled through the solution to precipitate 2-thioHis. The reaction was cooled to room temperature and then chilled on ice to allow complete precipitation. The off-white precipitate was filtered out of the reaction and washed with cold deionized water and ethanol. The precipitate was dried under high vacuum to provide 5.04 g (26.9 mmol) of an off-white powder. The percent yield of this reaction varies between 40% and 50% in our hands, which is consistent with the findings of Erdelmeier and coworkers [36]. MS analysis revealed a peak at 188.1 *m*/*z*. ^1^H-NMR (D_2_O/DCl): δ 3.06–3.20 (2H, (3.06 dd) (3.20 dd)), 4.21 (1H, dd), 6.79 (1H, s); ^13^C-NMR (D_2_O/DCl): δ 25.32, 51.82, 115.96, 123.23, 156.49, 170.38.

### 2.3. Enzyme Production

For this study, we used mouse mitochondrial Sec-TrxR, which contains selenocysteine (Sec), and a Cys-ortholog enzyme from *Drosophila melanogaster* (DmTrxR). The production and purification of Sec-TrxR, DmTrxR, and mutant versions have been reported previously [37,38].

### 2.4. MS Analysis of EGT Oxidized by H_2_O_2_

MS analyses of 10 mM 2-thioHis with 5 mM H_2_O_2_ and 10 mM EGT with 5 mM H_2_O_2_ solutions were obtained to determine what oxidized species were present. The samples were diluted 10-fold with a 50 mM ammonium bicarbonate, 0.1% formic acid solution, then injected immediately into a Thermo Scientific™ Q Exactive™ Hybrid Quadrupole-Orbitrap Mass Spectrometer set to positive electrospray ionization (ESI). The syringe pump used was a Chemyx Fusion 101 (Stafford, TX, USA). For each sample, 12–14 scans were averaged. The scan parameters were as follows: scan range, 70–1500 *m*/*z*; resolution, 70,000; polarity, positive; number of microscans, 3; automatic gain control (AGC) target, 1 × 10^6^; maximum inject time, 50 ms; flow rate, 10 μL/min. The heated electrospray ionization (HESI) source parameters were as follows: sheath gas flow rate, 15; auxiliary gas flow rate, 5; sweep gas flow rate, 0; spray voltage, 3.50 kV; capillary temperature, 250 °C; S-lens RF-level, 50.0; auxiliary gas heater temperature, 100 °C.

### 2.5. Sec-TrxR Activity Assay with ESSE

A 10 mM solution of EGT was prepared in 0.1 M potassium phosphate, pH 7.0. To this solution, 5 mM H_2_O_2_, freshly prepared in deionized water, was added to form the disulfide ESSE. This reaction was allowed to incubate at room temperature in the dark for 10 min. The activity of Sec-TrxR with ESSE was measured by monitoring the decrease in NADPH absorbance at 340 nm over 2 min using an extinction coefficient of 6220 M^−1^ cm^−1^. A 20 mM stock solution of NADPH was prepared in deionized water and frozen in aliquots to be used for activity assays. The ability of Sec-TrxR to reduce ESSE was measured in 500 μL assays with 200 μM NADPH and oxidized ESSE concentrations ranging from 5 to 500 μM. All assays were initiated with the addition of 10 nM Sec-TrxR enzyme. Controls were performed with corresponding concentrations of H_2_O_2_ and 2-thioHis or EGT alone using the same NADPH and enzyme concentrations. Parallel experiments were performed with 2-thioHis and selenoneine at concentrations ranging from 0.1 to 2 mM and 0.1 to 4 mM, respectively. The assays with 2-thioHis and selenoneine were initiated with 5 nM Sec-TrxR. Selenoneine exists as the diselenide in aqueous solution at neutral pH at room temperature, so it was not oxidized with H_2_O_2_ [13]. All assays were performed in triplicate, and the average of these trials was reported.

Additional assays were performed with Sec-TrxR, Trx, and ESSE. These assays contained 10 μM Trx, 200 μM NADPH, 10 nM Sec-TrxR, and ESSE in concentrations ranging from 5 to 300 μM. The activity of Sec-TrxR with 10 μM Trx was subtracted from all data points to remove background activity.

### 2.6. GR/GSH Activity Assay with ESSE

These assays were performed using the same procedure as for Sec-TrxR with ESSE, except GR/GSH were used in place of Sec-TrxR. These assays contained 200 μM GSH, 0.60 nM GR, and 200 μM NADPH. The concentration of ESSE was varied from 10 μM to 1 mM. The decrease in NADPH absorbance at 340 nm was monitored for 2 min. The assay was performed in triplicate, and the results were averaged. Analogous experiments were performed with 2-thioHis disulfide in the concentration range of 50 μM to 4 mM and were initiated by the addition of 0.75 nM GR. The background activity of GR with 200 μM GSH was subtracted from all data points.

### 2.7. NMR Experiments with EGT and H_2_O_2_

^1^H-NMR was used to quantify desulfurization of EGT upon treatment with H_2_O_2_. One NMR sample was prepared with 10 mM EGT in D_2_O, and one was prepared with 10 mM EGT and 5 mM H_2_O_2_ in D_2_O. Quantitative ^1^H-NMR was obtained on a Bruker Advance III HD 500 MHz NMR spectrometer for both samples. To obtain quantitative spectra, a D1 of 69 s was used based on a T1 relaxation value of 14.42 s for the C2 proton of hercynine; 2 dummy scans and 8 scans were obtained over a time period of ~12 min. The H_2_O_2_ was added to the second sample exactly 10 min before recording NMR spectra.

### 2.8. p-Nitrosodimethylaniline (RNO) Bleaching

Singlet oxygen was produced using the photosensitizer rose bengal in a 90% D_2_O solution by shining light on the solution following the general methods described by Kochevar and Redmond and Herman and Neal [39,40]. Conditions for producing ^1^O_2_ and oxidizing EGT were optimized using RNO bleaching to monitor production/scavenging of ^1^O_2_ using the procedure from Herman and Neal and Kraljić and El Mohsni [40,41]. Samples with 10 μM rose bengal and 8 mM imidazole and 10 μM rose bengal alone were prepared in 20 mM potassium phosphate-buffered 90% D_2_O, pH 7.0. To both samples, 50 μM RNO was added. An initial absorbance scan of both samples was taken, noting the absorbance of RNO at 440 nm. Both samples were then incubated under light, and absorbance scans were obtained every 3–4 min for 10 min. In addition, samples of 1 mM 2-thioHis with 10 μM rose bengal in D_2_O were placed on ice and irradiated with light for 20 min while monitoring changes in absorbance of 2-thioHis at 255 nm.

### 2.9. MS Analysis of EGT Oxidized by ^1^O_2_

MS analysis of the irradiated rose bengal/2-thioHis and rose bengal/EGT samples were completed to determine what oxidized species were present. MS analysis of oxidized 2-thioHis and EGT samples with rose bengal in D_2_O was completed within 30–45 min of irradiating the samples to avoid significant decomposition of any oxidation products. For these analyses, an Applied Biosystems QTrap 4000 hybrid triple-quadrupole/linear ion trap LCMS (SciEx, Framingham, MA, USA) was used. Positive electrospray ionization (ESI) was used as the ionization source. Samples were diluted with deionized water and directly infused via a Harvard Apparatus model 22 syringe pump (Harvard Apparatus, Holliston, MA, USA) at 5 µL/min into an isocratic (50% water and 50% acetonitrile with 0.1% formic acid) mobile phase flow from a Shimadzu Prominence high-performance liquid chromatography (HPLC) system (Shimadzu Scientific Instruments, Columbia, MD, USA). Mobile phase flow was maintained at 100 µL/min. Source temperature was maintained at 400 °C. Nitrogen was used for the sheath gas, auxiliary gas, and curtain gas. Sheath gas (GS1) flow was set at 40, auxiliary gas flow (GS2) at 50, curtain gas flow (CUR) at 30, and the declustering potential (DP) was set to 50. The mass spectrometer was operated in single quadrupole mode, scanning from *m*/*z* 100 to 1000.

### 2.10. Sec-TrxR Activity Assay with EGT Oxidized by ^1^O_2_

Singlet oxygen was produced using the procedure described in Section 2.8 with some alterations as described below. Stock solutions of 2 mM rose bengal in ethanol, and 10 mM EGT or 2-thioHis in deionized H_2_O were prepared and stored in the dark on ice and at room temperature, respectively. A 1 mL sample was prepared with 10 μM rose bengal and 1 mM EGT or 2-thioHis in D_2_O in a small glass test tube. This sample was incubated under a visible light source with a UV filter for 20 min. Following this incubation, the activity of Sec-TrxR with ^1^O_2_ oxidized 2-thioHis and EGT was measured by monitoring the decrease in NADPH absorbance at 340 nm over 2 min using an extinction coefficient of 6220 M^−1^ cm^−1^. Aliquots of oxidized EGT or 2-thioHis were added to 500 μL assays in 0.1 M potassium phosphate buffer, pH 7.0 with 1 mM EDTA and 200 μM NADPH. Assays were initiated with 20 nM Sec-TrxR. To generate an activity curve for ^1^O_2_ oxidized EGT, 2 min assays were performed for concentrations ranging from 10 to 240 μM of the rose bengal/EGT solution. Analogous experiments were performed with 2-thioHis. The irradiated rose bengal/2-thioHis, or EGT solutions, were kept in amber tubes and either used fresh in assays or kept on ice and used within 30–45 min of irradiation by light as the oxidation products were not very stable at room temperature. Controls were performed with rose bengal and 2-thioHis alone added to Sec-TrxR and NADPH to ensure any observed activity was enzyme catalyzed.

### 2.11. MS Analysis of ^1^O_2_-Oxidized EGT following Reduction with Sec-TrxR

To confirm that Sec-TrxR could reduce ^1^O_2_-oxidized EGT, MS analysis was performed on a sample with 1 mM EGT and 10 μM rose bengal in 90% D_2_O, irradiated with light for 20 min, then diluted with pH 8.0 ammonium bicarbonate buffer and reacted with NADPH and Sec-TrxR for 10 min at 37 °C in a final volume of 1.262 mL. The final concentrations of compounds in the sample were as follows: 792 μM EGT, 7.92 μM rose bengal, 792 μM NADPH, and 190 nM Sec-TrxR in 80 mM ammonium bicarbonate buffer, pH 8.0. After incubation of the sample for 10 min, the sample was run through an Amicon^®^ Ultra 30K centrifugal filter unit (Millipore, Burlington, MA, USA), spinning the sample at 14,000× *g* for 5 min, to separate the enzyme from the rest of the sample. The flow-through from the filtration (no enzyme) was analyzed by direct-inject positive ESI MS. For these analyses, an Applied Biosystems QTrap 4000 hybrid triple-quadrupole/linear ion trap liquid chromatograph-mass spectrometer (SciEx, Framingham, MA, USA) was used. The same method and parameters described in Section 2.9 were used for this sample, except the mass range was altered to *m*/*z* 100 to 600. A control experiment was run using the same procedure outlined above, except 62 μL of deionized water was added in place of Sec-TrxR and NADPH to achieve the same final EGT and rose bengal concentrations. Following ultrafiltration, the flow-through from the control sample was analyzed using a Waters Xevo G2-S Q-TOF LCMS (Waters, Milford, MA, USA).

### 2.12. Selenium Dependency of ESSE Recycling by TrxR Experiments

The selenium dependency of reduction of ESSE by TrxR was determined by testing the ability of TrxR∆3 (truncated enzyme missing three C-terminal amino acids), TrxR-GCCG (Sec→Cys mutant), and DmTrxR to catalyze the reduction of ESSE. Assays were performed in 0.1 M potassium phosphate buffer containing 1 mM EDTA with 200 μM NADPH and 400 μM ESSE. The assays were initiated with 300 nM TrxR∆3, 114 (pH 7) or 68 (pH 8) nM TrxR-GCCG, and 80 nM DmTrxR. All enzymes were tested at both pH 7.0 and pH 8.0. The decrease in absorbance at 340 nm was monitored for 2 min for each sample.

## 3. Results and Discussion

### 3.1. Enzymatic Reduction of ESSE with TrxR

We generated ESSE by the reaction of EGT with H_2_O_2_ (2:1) in 0.1 M potassium phosphate, pH 7.0. The disulfide of 2-thioHis was generated in an identical manner. We used 2-thioHis as a less expensive analog of EGT in order to experiment with the right conditions for generating various oxidized forms of EGT. Selenoneine does not exist in the reduced form and was provided as the diselenide form, and we, therefore, did not need to oxidize it further [13].

**Figure 2:**
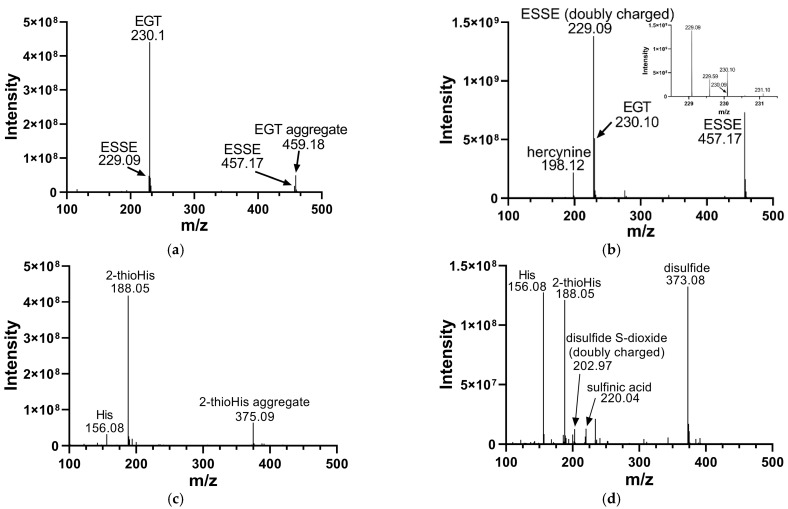
Mass spectra of EGT and 2-thioHis before and after oxidation with H_2_O_2_. (**a**) Mass spectrum of EGT freshly prepared in deionized water. (**b**) Mass spectrum of EGT/H_2_O_2_ (2:1) in deionized water following a 10 min incubation with H_2_O_2_. The inset is a close-up of *m*/*z* 229 and *m*/*z* 230. (**c**) Mass spectrum of 2-thioHis freshly prepared in deionized water. (**d**) Mass spectrum of 2-thioHis/H_2_O_2_ (2:1) in deionized water following a 10 min incubation with H_2_O_2_.

We analyzed the products of these reactions by electrospray MS. The results of these analyses are shown in Figure 2. Oxidation of EGT with H_2_O_2_ shows more complete conversion to the disulfide form in comparison to 2-thioHis, with somewhat less desulfurization as shown by the presence of hercynine in the MS (Figure 2b). Hercynine is the name for the desulfurized form of EGT. Desulfurization results from overoxidation of ESSE, as previously discussed by Servillo and coworkers [15]. The overoxidation products of 2-thioHis that we detected are His, the sulfinic acid, and the disulfide-S-dioxide. One explanation for the differences in oxidation products between EGT and 2-thioHis is that oxidation of EGT results in a more hindered disulfide, which may be less susceptible to hydrolysis and overoxidation.

Early studies of the oxidation of EGT to ESSE reported that ESSE was only stable at acidic pH [42]. However, our data shows that ESSE is formed easily in water or neutral pH in the presence of EGT and H_2_O_2_ in a 2:1 ratio and is stable for at least several hours. This agrees with more recent data from Servillo and coworkers, who showed that ESSE rapidly forms in the presence of various oxidants and then decomposes over a 36 h period [15]. They showed that oxidation of EGT by various oxidants proceeded to ESSE as an intermediate, which then underwent oxidative breakdown to the sulfinic acid and ESH when EGT and oxidant were combined in a 1:1 ratio [15]. As we combined EGT and oxidant in a 2:1 ratio, the lifetime of the disulfide should be considerably longer, as supported by our data.

**Figure 3:**
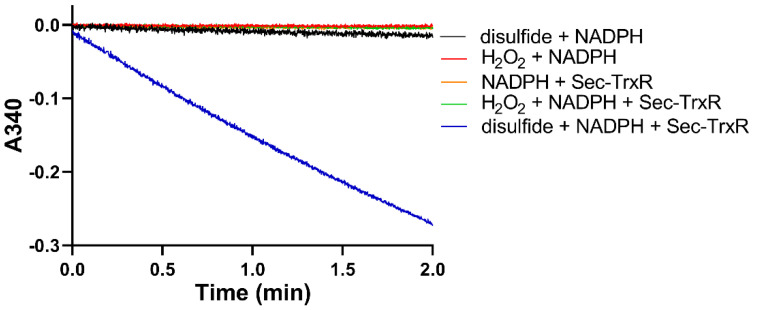
Consumption of NADPH by Sec-TrxR is stimulated by the presence of 2-thioHis disulfide, but not in its absence. The blue line shows that NADPH is only consumed when 2-thioHis disulfide is added to the assay. Control conditions are described in the legend at the right of the figure.

After generating the disulfide in situ by addition of H_2_O_2_, we performed enzyme assays with Sec-TrxR by adding aliquots of the reaction mixture to an assay containing enzyme and NADPH. To ensure the activity that we were detecting was due to reduction by Sec-TrxR, we performed various controls without enzyme (or enzyme plus H_2_O_2_), as shown in Figure 3. As is shown in the plot, activity is only present when the assay contains substrate (disulfide of 2-thioHis), NADPH, and enzyme. We subsequently repeated the same experiment by oxidizing EGT with H_2_O_2_. An important control in Figure 3 is the assay of Sec-TrxR in the presence of 1 mM H_2_O_2_ (green trace), which shows no activity. Even though we have previously reported that H_2_O_2_ is a “substrate” for Sec-TrxR, the *K*_M_ for H_2_O_2_ is ~260 mM, with detectable activity only occurring at concentrations above 5 mM [38]. Under the conditions of our assay (1 mM), the presence of H_2_O_2_ does not contribute to the observed activity. This assay was also performed either in the presence or absence of 10 μM *E. coli* Trx. We assayed selenoneine without the addition of H_2_O_2_ because it autoxidizes to the diselenide form.

The Michaelis–Menten plots of these enzyme assays are shown in Figure S1 of the Supporting Information. The addition of 10 μM *E. coli* Trx to the assay containing ESSE resulted in a 3-fold lower *K*_M_ value as well as a lower *k*_cat_. This assay, in part, reflects in vivo conditions where Trx would be present. We chose a concentration of 10 μM Trx for the assay because higher values resulted in activity that was non-linear in the spectrophotometric assay even though 10 μM is less than the *K*_M_ for *E. coli* Trx. While the data in Figure 3 clearly shows that Sec-TrxR can directly reduce ESSE, Sec-TrxR and Trx can work together to reduce substrates. In such cases, Trx(SH)_2_ can reduce ESSE to 2EGT, forming Trx(S–S). Sec-TrxR then reduces the Trx(S–S) back to Trx(SH)_2_ to restart the cycle. This explains why there is a 3-fold lower *K*_M_ value when Trx is added to the assay because Trx has a higher binding affinity for Sec-TrxR compared to ESSE.

**Figure 4:**
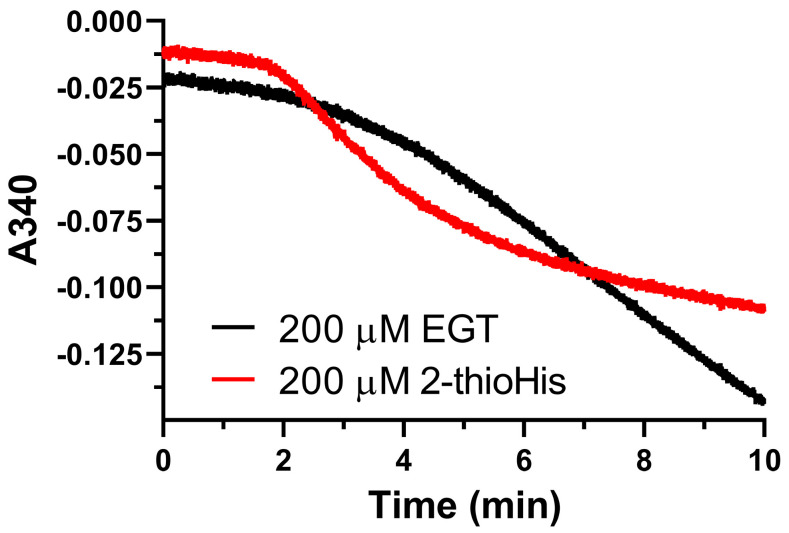
Unoxidized EGT and 2-thioHis stimulate consumption of NADPH by Sec-TrxR. Absorbance versus time plot showing consumption of NADPH by Sec-TrxR over a 10 min time period in the presence of EGT or 2-thioHis, but in the absence of oxidant.

One interesting phenomenon that we observed was that we could detect NADPH consumption by Sec-TrxR when either EGT or 2-thioHis was present in the assay, but in the absence of H_2_O_2_ after a long lag phase, as shown in Figure 4. The standard method for assaying TrxR is to observe the consumption of NADPH in the presence of substrate for the first minute of the assay. We have typically observed 2 min of data in all of our studies on TrxR. When performing control experiments during this time interval, we observed no consumption of NADPH when either 2-thioHis or EGT was present in the assay in the absence of oxidants such as H_2_O_2_. Serendipitously, we made the observation that the addition of either EGT or 2-thioHis to the assay in the absence of H_2_O_2_ resulted in consumption of NADPH after about 3.5 min. This activity is equivalent to ~20% of the activity compared to when H_2_O_2_ is added to the assay. One explanation is that EGT and 2-thioHis slowly autoxidize to the disulfide after about 3 min. However, it has been reported that EGT resists autoxidation due to it being present largely as the thione and not the thiol [6,8,15]. Alternatively, TrxR is known to have NADPH oxidase activity that produces H_2_O_2_ in the absence of its cognate substrate, Trx [43]. The observed lag phase is consistent with this hypothesis as it would take a small amount of time to produce enough H_2_O_2_ to oxidize the EGT or 2-thioHis in solution. While this is one possible explanation, we cannot definitively explain this phenomenon. However, our MS data makes it clear that the addition of H_2_O_2_ to EGT or 2-thioHis results in the formation of the disulfide, which is a substrate for Sec-TrxR.

### 3.2. Enzymatic Reduction of ESSE with GR/GSH

The other major antioxidant system in mammalian cells is the glutathione system. There are several enzymes of the glutathione system, including GR, glutaredoxin, and glutathione peroxidase [35]. The function of GR is to recycle oxidized glutathione (GSSG) to reduced glutathione (GSH) [35]. GSH is a general antioxidant in the cell and can function to reduce protein disulfide bonds and other low-molecular-weight disulfides such as ESSE [35,44]. Since mammalian GR is highly homologous to TrxR but lacks the Sec-containing C-terminal redox center, we decided to test whether GR alone or in combination with GSH could reduce ESSE [45,46].

**Figure 5:**
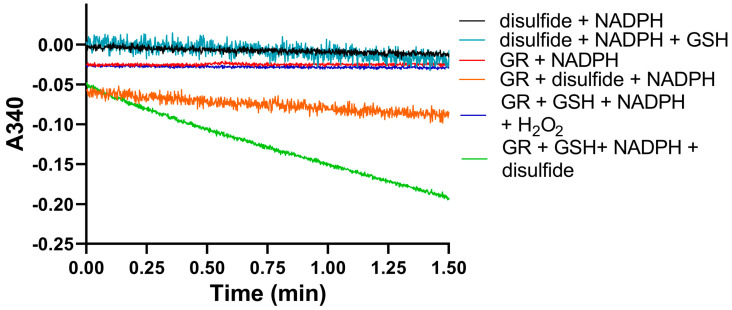
Consumption of NADPH by GR is stimulated by the presence of 2-thioHis disulfide and GSH. The green line shows that NADPH is only consumed when 2-thioHis disulfide and GSH are added to the assay. Control conditions are described in the legend at the right of the figure. As shown by the orange line, GR shows very poor activity toward the disulfide form of 2-thioHis when GSH is not present.

**Figure 6:**
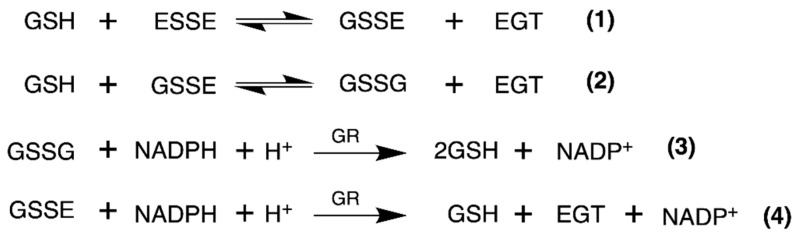
Equations that describe the mechanism of reduction of ESSE by GR. The figure and equations are adapted from the work of Eyer and Prodhradský, who elucidated the mechanism of reduction of 5,5′-dithiobis-(2-nitrobenzoic acid) (DTNB) by GR [47].

We used identical conditions for generating the disulfide forms of EGT and 2-thioHis in our enzymatic assays with GR or GR/GSH as we used for our assays with Sec-TrxR. In order to demonstrate that the consumption of NADPH that we observed in these experiments was due to enzymatic activity, we performed identical control experiments as was performed in our TrxR assays, starting with the disulfide of 2-thioHis. The result of this experiment is shown in Figure 5 and shows that the generated disulfide is a very poor substrate for GR itself. This is unlike Sec-TrxR. However, the addition of GSH to the assay greatly stimulates enzymatic activity. The reason for this is that GSH can attack the disulfide substrate (either ESSE or 2-thioHis disulfide) and form a mixed disulfide, as shown by Equation (1) in Figure 6. The mixed disulfide can be attacked by another equivalent of GSH to form GSSG (Equation (2)). The resulting GSSG can then be reduced by GR (Equation (3)). Another possibility is that GR efficiently reduces the mixed disulfide, as described by Equation (4). Comparing the kinetic parameters for the reduction of ESSE and 2-thioHis disulfide by GR provides insight into which mechanism is correct, as discussed below.

**Figure 7:**
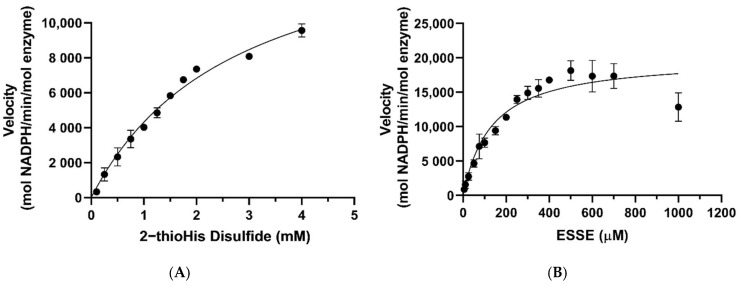
Michaelis–Menten curves for 2-thioHis-disulfide and ESSE with GR/GSH. (**A**) Activity of GR/GSH with 2-thioHis oxidized by H_2_O_2_. (**B**) Activity of GR/GSH with EGT oxidized by H_2_O_2_.

The Michaelis–Menten plots for the reduction of ESSE and 2-thioHis disulfide by the GR/GSH system are shown in Figure 7. One notable feature is that ESSE appeared to have an inhibitory effect on GR at high substrate concentrations, unlike 2-thioHis-disulfide, causing the activity to level off and then go back down (Figure 7B). The different activity patterns observed between 2-thioHis-disulfide and ESSE were unexpected. The only difference between the molecules is that the amine of EGT is trimethylated, so it always carries a positive charge that is spread over the methyl groups. While this difference should have little impact on the redox chemistry of the two compounds, it provides an important clue in differentiating whether GR is reducing GSSG in the assay as described by Equation (3) or whether GR is reducing the mixed disulfide (GSSE) as described by Equation (4).

As listed in Table 1, the *k*_cat_ values for the reduction of 2-thioHis disulfide and ESSE by GR/GSH are very similar, 16,425 min^−1^ and 20,035 min^−1^, respectively. However, the *K*_M_ values diverge by ~22-fold. If GSSG is being formed in the reaction of both disulfide substrates as described by Equation (3), one would expect very similar *K*_M_ values. If the mixed disulfide is the substrate for GR, on the other hand, as described by Equation (4), different *K*_M_ values are expected because one mixed disulfide has a trimethylated amine and the other one does not. The very different *K*_M_ values argue strongly that the mixed disulfide is the substrate for GR. Another piece of evidence for this contention is that the concentration of GSH in the assay was kept constant at 200 μM. As shown by the data in Figure 7A, all of the assay points for 2-thioHis disulfide (except for two) were performed at a concentration of 500 μM or higher. At 500 μM 2-thioHis disulfide, 1 mM GSH is required to form GSSG, and this is 5-fold more than is actually present in the assay. The situation is amplified at the highest substrate concentration (4 mM), requiring 8 mM GSH in order to form GSSG.

Other support for the mixed disulfide being the substrate comes from the literature. Eyer and Prodhradský investigated the mechanism of reduction of DTNB by GR/GSH and found that all of the GSH in the assay was consumed as described in Equation (1) of Figure 6 [47]. They concluded from their analyses that the mixed disulfide between GSH and 2-nitro-5-mercapto-benzoic acid (TNB) was the substrate in their assay using GR/GSH. DTNB is a suitable model for ESSE as both disulfides are highly reactive due to the polarization of the disulfide bond caused by electron-withdrawing groups on each sulfur atom. Another example from the literature is from Gruhlke and coworkers, who showed that the mixed disulfide between allicin and GSH was a very suitable substrate for GR [48]. Based on our analysis above and literature precedent, we conclude that the mixed disulfide (either GSSE or GSS2TH) is the substrate for GR in our assay.

We must strongly emphasize that our analysis above is predicated on the fact that the equilibrium constant for (1) is much greater than that of (2). If this is not the case and the equilibrium constants are similar such that the two equilibria are in competition, then our derived values of *K*_M_ and *k*_cat_ in Table 1 are meaningless.

Based on the structural difference between the two different types of mixed disulfides described above, we can offer a more specific reason for the inhibition observed in Figure 7B. The reason for the decrease in activity is that half of the disulfide that contains the positively charged trimethylamine of EGT could potentially mimic NADP^+^ and disrupt the π-stacking and cation-π interactions between NADP^+^ and FADH_2_ at the NADPH binding site of GR [49,50,51,52]. This type of interaction is missing in the case of 2-thioHis. EGT is more likely to participate in stronger cation-π interactions than 2-thioHis due to the positively charged trimethylated amine group hosting a very stable cation [49,50,51]. Intracellular stores of EGT are typically kept in the low millimolar range [1,5,53]. A reduction in activity was not seen until a concentration of 1 mM ESSE (2 mM EGT) was reached, suggesting that the disruption of the π-stacking interaction by EGT occurs only at high physiological concentrations. Interestingly, we noticed that when we followed the reaction with ESSE over a long time period (10 min), the activity increased (Figure S2 of the Supporting Information). A possible explanation for this lag phase is that once some of the disulfide is reduced, the inhibition is diminished.

A summary of the data for both TrxR and GR/GSH is given in Table 1. A comparison of ESSE as the substrate for both systems without Trx shows a ~3-fold lower *K*_M_ value and a ~7-fold higher *k*_cat_ for GR/GSH. As a result, the catalytic efficiency of GR/GSH is ~20-fold higher than TrxR alone. However, when comparing the GR/GSH system with the TrxR/Trx system, the difference in *k*_cat_ values is ~10-fold, and the *K*_M_ values are similar. This results in a ~10-fold higher catalytic efficiency for the GR/GSH system. However, the catalytic efficiency of both systems may be similar at a concentration of Trx that is close to the *K*_M_ (physiological concentration), but we were unable to make this measurement in vitro. Our in vitro study cannot distinguish which antioxidant system is more important in vivo, and this remains a question for future investigation. However, TrxR has the advantage that it can directly reduce ESSE, and GR cannot.

Selenoneine is the selenium-analog of EGT and was discovered in 2010 in the blood of tuna fish [13,14]. Similar in function to EGT, selenoneine possesses powerful antioxidant properties in vitro and has also been shown to detoxify heavy metals such as mercury in vivo [14,54,55]. Humans obtain selenoneine by consuming fish and accumulating it in their cells via the cation/carnitine transporter OCTN1 [14,54,55]. It is interesting to note that selenoneine binds less tightly to Sec-TrxR, as reflected by a 5-fold higher *K*_M_ value in comparison to ESSE but is only turned over 2-fold faster. As a result, the catalytic efficiency is ~2-fold lower.

We must note that the activity of Sec-TrxR and GR/GSH toward ESSE is potentially higher than reported in Table 1 because MS analysis of the oxidation of EGT with H_2_O_2_ (Figure 2) revealed the presence of hercynine. This means that the calculated amount of disulfide substrate added to our assays is somewhat less than is reported in the plots of activity versus substrate concentration shown in Figure S1 of the Supporting Information. Because the substrate was produced by the reaction of EGT with H_2_O_2_ immediately before assaying, we have no way of correcting this small error. To determine if significant desulfurization occurred, ^1^H-NMR experiments were performed with EGT in D_2_O and EGT in D_2_O incubated with H_2_O_2_ for 10 min. The results of these experiments are shown in Figure S3 of the Supporting Information. The amount of desulfurization was calculated to be 3% in the oxidized sample compared to 2% in the unoxidized EGT sample, leading us to conclude that the amount of desulfurization that occurs as a result of oxidation of EGT with H_2_O_2_ is not extensive in the time-frame of the enzyme kinetics assays.

While we used H_2_O_2_ as the oxidant to generate ESSE in this study as a matter of convenience, Servillo and coworkers showed that several other biologically relevant oxidants oxidized EGT to ESSE at a much faster rate, especially HOCl [15]. HOCl is produced in neutrophils and monocytes as part of the innate immune system. We note that Servillo and coworkers not only studied the formation and decomposition of ESSE in vitro but demonstrated that ESSE forms in neutrophils and endothelial cells from various different oxidants, especially HOCl and superoxide [15,16]. Thus, there are multiple pathways for the biological formation of ESSE in animal cells, which could then be reduced back to EGT by Sec-TrxR or GR/GSH. We think it is logical that the cell would have a way to recycle oxidized forms of EGT, thus conserving it since the cell has a specific transporter for this dietary-derived nutrient.

### 3.3. Activity of Sec-TrxR toward 2-ThioHis and EGT Oxidized with ^1^O_2_

Singlet oxygen is a biologically significant oxidant produced in humans and other animal species [24,56,57,58,59,60,61]. It is produced by cells of the immune system such as macrophages and neutrophils, where it is used to kill bacteria [62,63,64]. Singlet oxygen is generated by the enzymatic action of myeloperoxidase and superoxide dismutase, respectively, in these cell types [62,63,64]. It is notable that EGT, like vitamin C, is accumulated in these cells of the immune system [6,65,66]. We also note that EGT is a very good quencher of ^1^O_2_, with a higher rate constant than GSH [24,67]. It has also been shown to be a superior quencher of ^1^O_2_ compared to Asc [20]. Singlet oxygen can also be produced in the skin and eye due to the presence of photosensitizers that catalyze the conversion of ^3^O_2_ (ground state) to ^1^O_2_ (excited state) [57,58]. In addition, the reaction of sunlight with protoporphyrin IX, the iron-free precursor of heme, produces ^1^O_2_ in erythropoietic cells [68].

**Figure 8:**
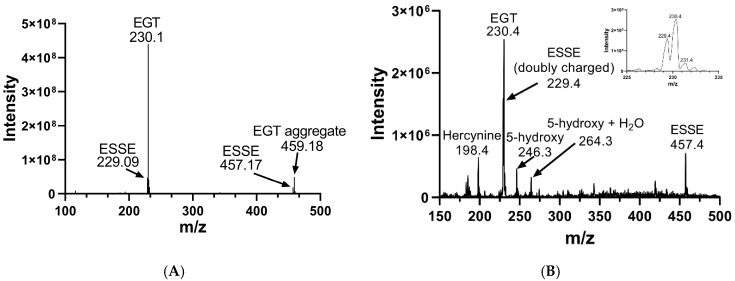
Mass spectra of EGT before and after oxidation with ^1^O_2_. (**A**) MS of EGT in deionized water in the absence of ^1^O_2_. (**B**) MS of EGT oxidized with ^1^O_2_ (rose bengal + light).

We oxidized EGT using ^1^O_2_ that was generated by using the photosensitizer rose bengal in the presence of a visible light source in 90% D_2_O for 20 min. The decision to use D_2_O instead of water or buffer was made because the lifetime of ^1^O_2_ is significantly longer in D_2_O due to the shifted vibrational frequencies of the D–O bonds, allowing for more extensive oxidation of our compounds [69,70]. We then analyzed the products of this reaction using MS, which is shown in Figure 8. MS analysis of EGT oxidized with ^1^O_2_ showed the trace presence of the 5-oxo species at 244 *m*/*z* as well as the 5-hydroxy species at 246 *m*/*z* and ESSE at 229 *m*/*z* and 457 *m*/*z* (Figure 7B). We note that the 5-hydroxy species is equivalent to the 5-oxo species, with the 5-hydroxy form existing at low pH (as occurs in the electrospray) and the 5-oxo species occurring at neutral pH. MS analysis of 2-thioHis oxidized with ^1^O_2_ is shown in Figure S4 of the Supporting Information.

**Figure 9:**
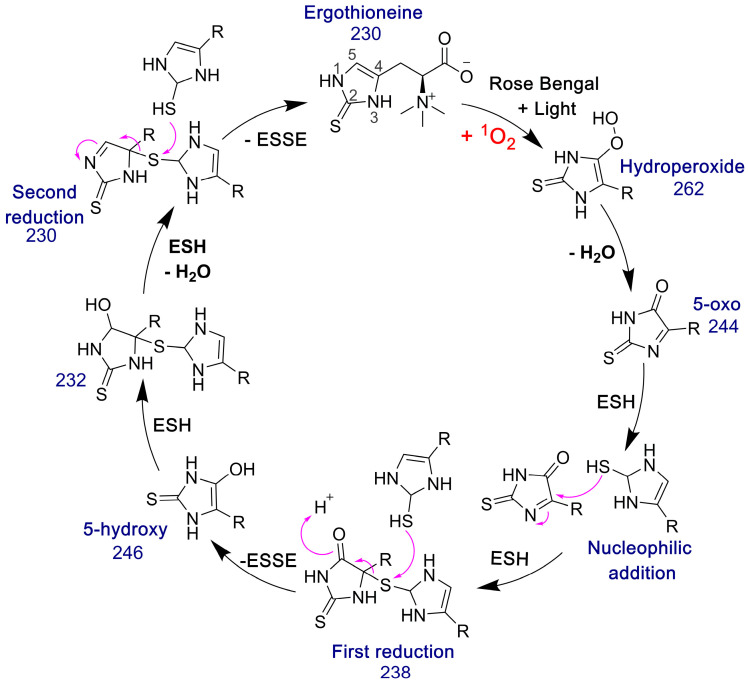
Pathway for the formation of ESSE from 5-oxo-EGT and ESH. The *m*/*z* values for each species are provided. This figure was adapted from the GSH/EGT cycle proposed by Gründemann and coworkers [20]. Note that R = −CH_2_CH(COO^−^)N^+^(CH_3_)_3_.

The 5-oxo form of EGT is formed by oxidation of EGT with ^1^O_2_ by reaction of this excited form of oxygen with the C5 position to produce a hydroperoxide intermediate as shown in Figure 9, which then eliminates H_2_O to produce the 5-oxo form. This was shown recently by Gründemann and coworkers [20]. ESSE can then form from the 5-oxo compound if another molecule of ESH attacks at the 4-position, forming a thioether intermediate. This step is followed by attack of a second molecule of ESH onto the sulfur of the thioether, resulting in the formation of ESSE. This is identical to the mechanism for EGT recycling by GSH proposed by Gründemann and coworkers resulting in the formation of GSSG [20]. The complete reduction of the 5-oxo form of EGT requires four equivalents of ESH to reduce 5-oxo-EGT to EGT, resulting in two equivalents of ESSE. The MS samples were run 30–45 min after completion of the reaction allowing for plenty of time for the formation of ESSE and other reduction products, which accounts for the significant amount of ESSE detected by MS, as shown in Figure 8B. We also note that the second reduction product has an *m*/*z* value of 230 for the doubly charged species, which is the same *m*/*z* value for singly charged EGT. Due to the resolution of our MS spectrum, we cannot determine if the 230 *m*/*z* peak is a mixture of these two species or not.

We confirmed that ^1^O_2_ was generated in our reaction by measuring the change in absorbance of RNO after exposure to visible light in the presence of rose bengal for 10 min. Imidazole and its derivatives will react with ^1^O_2_ and form a transannular peroxide, which then reacts with RNO resulting in “RNO bleaching”, which can be detected by a change in absorbance at 440 nm [40,41]. In the absence of imidazole the UV-Vis spectrum showed little change at 440 nm, indicating very little oxidation of RNO, as expected. However, when we added imidazole to the same reaction, a large change at 440 nm was observed, indicating oxidation by ^1^O_2_. An additional control was performed with rose bengal and 2-thioHis, monitoring the change in absorbance of 2-thioHis at 255 nm over a 20 min incubation. These control reactions are shown in Figures S5 and S6 of the Supporting Information.

**Figure 10:**
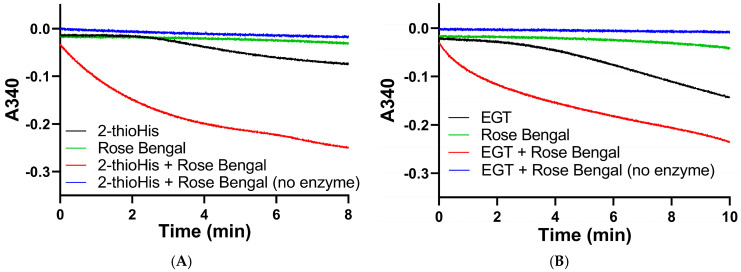
Absorbance versus time curves for the reaction of TrxR with EGT or 2-thioHis oxidized by ^1^O_2_. (**A**) Change in absorbance over time plot for oxidized 2-thioHis and controls. (**B**) Change in absorbance over time plot for oxidized EGT and controls. All samples have NADPH added to them. All samples have Sec-TrxR added to them except the blue lines.

We subsequently assayed the solutions of 2-thioHis and EGT that were oxidized by the ^1^O_2_ that was generated from the photocatalytic reaction of rose bengal and ^3^O_2_ in the presence of visible light. The assay was performed by adding aliquots of the oxidized compounds immediately after exposure to 20 min of visible light to an assay mixture that contained 200 μM NADPH, potassium phosphate buffer, pH 7.0, and 20 nM Sec-TrxR. The result of this assay shows that NADPH was rapidly consumed in the presence of Sec-TrxR, but not when the enzyme was absent (compare red and blue traces in Figure 10A,B). When rose bengal is omitted from the reaction, there is very little consumption of NADPH for the first 3–4 min of the reaction. However, as the reaction progressed over a longer time period, we noticed consumption of NADPH that was less than when ^1^O_2_ was present due to the photocatalytic reaction of rose bengal and ^3^O_2_, but more than when the enzyme was absent (compare black and blue traces in Figure 10A,B). This same phenomenon was observed previously in the oxidation of 2-thioHis/EGT by H_2_O_2,_ as shown in Figure 4. This apparent oxidation of 2-thioHis/EGT in the absence of added external oxidant is potentially explained by the NADPH oxidase activity of Sec-TrxR or by autoxidation of 2-thioHis/EGT in solution that is possibly catalyzed by trace metals in the solution. However, we cannot definitively explain this observation at this time. A further control was performed in which 2-thioHis/EGT was omitted from the reaction, but rose bengal, NADPH, and enzyme were all present. No consumption of NADPH was observed (green trace in Figure 10). This shows that rose bengal or an oxidized form of rose bengal is not a substrate for Sec-TrxR.

As evidenced by the Michaelis–Menten plots in Figure S7 of the Supporting Information and the kinetic data summarized in Table 2, Sec-TrxR displayed better saturation toward aliquots of the solution of EGT that was oxidized by ^1^O_2_ compared to 2-thioHis resulting in a 2.5-fold lower *K*_M_ value, but a ~4-fold lower *k*_cat_. The small differences in kinetic parameters may be attributed to greater steric hindrance in the oxidized form of EGT due to the trimethylated amine. We must note that our MS analysis shows that solutions of 2-thioHis or EGT that are oxidized with ^1^O_2_ are a mixture of the 5-oxo and 5-hydroxy forms as well as the disulfide form, which we have shown is also a substrate for Sec-TrxR. Thus, our observed values of *k*_cat_ and *K*_M_ do not reflect the true values for these parameters.

**Figure 11:**
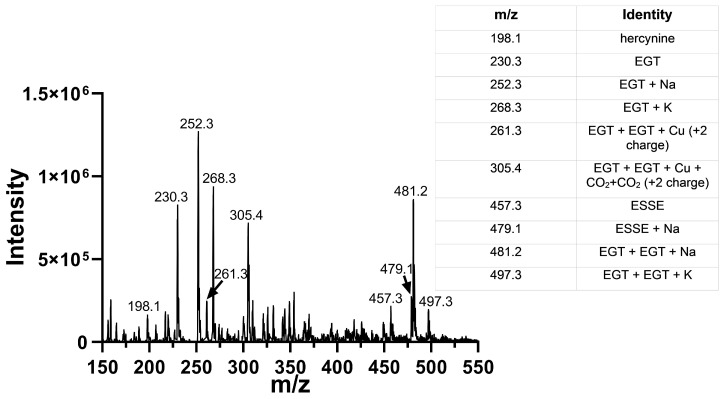
Mass spectra of ^1^O_2_-oxidized EGT followed by immediate addition of Sec-TrxR and NADPH. Comparison of the data above with the data in Figure 8B shows the disappearance of the peaks corresponding to 5-oxo-EGT and 5-hydroxy-EGT. Our interpretation is that Sec-TrxR can directly reduce the 5-oxo and 5-hydroxy forms back to EGT.

In order to verify that the activity we measured in our assay was at least in part due to the ability of Sec-TrxR to reduce the 5-oxo form of 2-thioHis/EGT, we added 200 μL of 500 mM ammonium bicarbonate, pH 8.0, to 1 mL of the irradiated reaction mixture containing 10 μM rose bengal and 1 mM EGT in 90% D_2_O followed by addition of Sec-TrxR (190 nM) and NADPH (800 μM). This reaction was incubated for 10 min at 37 °C. The enzyme was removed by ultrafiltration, and the flow-through was submitted for MS analysis. The resulting MS data is shown in Figure 11.

This MS experiment showed that Sec-TrxR completely reduced the 5-oxo and 5-hydroxy forms of EGT, as evidenced by the disappearance of the peaks at *m*/*z* = 244, *m*/*z* = 246, and *m*/*z* = 264. There was almost complete reduction of the disulfide ESSE (compare Figure 8B with Figure 11). In fact, the resulting MS spectrum contains almost all reduced EGT as the sodium or potassium adducts or two EGT molecules aggregated together with sodium or potassium. This MS experiment provides direct evidence of the reduction of ESSE, 5-hydroxy-EGT, and 5-oxo-EGT by Sec-TrxR, confirming that the recycling of these substrates by Sec-TrxR is responsible for the activity seen in our enzyme kinetics assays. The reduction of 5-oxo-EGT by Sec-TrxR, prior to MS analysis, was performed in ammonium bicarbonate buffer pH 8.0, which is different from our spectrophotometric assays, which were performed in potassium phosphate buffer, pH 7.0. As a control, we measured the rate of reduction of ^1^O_2_-oxidized EGT by Sec-TrxR with spectrophotometry under the same conditions to ensure the enzyme had high activity in this buffer (Figure S8 of the Supporting Information). A control MS experiment was run to verify that 5-oxo-EGT, 5-hydroxy-EGT, and ESSE did not undergo decomposition due to ultrafiltration (Figure S9 of the Supporting Information). All three of these oxidized species were detected in the control sample, confirming that they were stable to ultrafiltration.

As alluded to earlier, our computed *k*_cat_ and *K*_M_ values for the 5-oxo form are most assuredly greatly underestimated because the measured activity is a combination of the reduction of the 5-oxo form, 5-hydroxy form, and the disulfide form. However, one relevant point of comparison would be the reduction of dehydroascorbate by Sec-TrxR since there is a degree of structural similarity between 5-oxo EGT and dehydroascorbate, as shown in Figure 1. May and coworkers reported a *k*_cat_ of 90 min^−^^1^ and a *K*_M_ of 2.5 mM for the reduction of dehydroascorbate by Sec-TrxR [27]. Thus, 5-oxo EGT is a better substrate for Sec-TrxR by a factor of ~100 as measured by *k*_cat_/*K*_M_.

As shown in Figure 1, the reduction of the oxidized forms of vitamin C by Sec-TrxR is analogous to the reduction of the oxidized forms of EGT by Sec-TrxR, with the exception of the disulfide form of EGT, which does not exist for ascorbate. The relationship in Figure 1 underscores the “vitamin-like” nature of EGT and the role of TrxR in recycling oxidized forms of ascorbate and EGT.

### 3.4. Selenium Dependence of the Reactions

After demonstrating that Sec-TrxR can reduce ESSE, we sought to determine if the reaction is selenium dependent. To test the selenium dependency of the reaction, EGT oxidized by H_2_O_2_ was used as a substrate for TrxR∆3 (truncated enzyme missing three C-terminal amino acids), TrxR-GCCG (Sec→Cys mutant of Sec-TrxR), and DmTrxR (a Cys-ortholog from *D. melanogaster*). We note that this study uses the mitochondrial TrxR, which allows for better comparison to the Cys-ortholog TrxRs in this study as previously discussed by us [71].

The results of these assays are summarized in Table 3. The truncated enzyme TrxR∆3 had no ability to reduce ESSE even at 300 nM enzyme concentration, confirming that a C-terminus with Cys or Sec is necessary to reduce ESSE. The percent difference in activity between Sec-TrxR and TrxR∆3 was 99.8–99.9%, and activity showed little dependence upon pH. The mutant enzyme TrxR-GCCG had slightly higher activity than TrxR∆3, although it was 98.4–99.1% lower than Sec-TrxR. DmTrxR had more activity than either of the Sec-TrxR mutants and showed a greater dependence upon pH than either TrxR∆3 or TrxR-GCCG. The activity of DmTrxR toward ESSE was 94–97% lower than Sec-TrxR. Sec-TrxR showed the greatest pH dependence, with the activity toward ESSE almost doubling at pH 8 compared to pH 7. This is somewhat surprising because we had expected the activity of DmTrxR to increase more at higher pH because this would increase the proportion of Cys-thiolate relative to Cys-thiol. In contrast, the active site Sec residue should exist as the selenolate at both pH 7 and pH 8 since the p*K*_a_ of Sec is ~5.2 [72]. A possible explanation is that ESSE breaks down at pH 8 to ESOH (the sulfenic acid) and EGT, similar to the breakdown of DTNB at alkaline pH. The ESOH form could be a better substrate for Sec-TrxR than ESSE, with Sec-TrxR converting ESOH to EGT and water.

## 4. Conclusions

This report introduces a new and biologically relevant substrate for mammalian Sec-TrxR. We have demonstrated the ability of Sec-TrxR to directly reduce the 5-oxo and disulfide forms of EGT, produced by reaction with biologically relevant oxidants in vitro. The reduction of these oxidized forms of EGT is far more efficient with Sec-TrxR than it is with TrxR mutants without Sec or with the Cys-ortholog DmTrxR, showing that selenium has a major role in catalyzing the reaction. While this report demonstrates that Sec-TrxR can reduce oxidized forms of EGT in vitro, it remains to be seen how important the thioredoxin system is to recycling EGT in vivo since the glutathione system most likely makes an important contribution as well. Sec-TrxR has an advantage over GR as it can reduce ESSE directly without the aid of an exogenous thiol. This report brings us one step closer to understanding the biological chemistry and relevance of EGT.

While our results show that ESSE and 5-oxo-EGT are substrates for Sec-TrxR, we were unable to show that similar to Asc•, ES• was a substrate for Sec-TrxR because our initial experiments involved the use of various metals such as Fe^3+^ to generate the radical and NADPH can directly reduce Fe^3+^ in the absence of enzyme leading to very high background activity [73]. Since ES• and Asc• are obviously analogous structures (Figure 1), and May and coworkers have previously demonstrated that TrxR can reduce Asc• [26], we hypothesize that TrxR should be able to reduce ES• as well. EGT is a powerful radical scavenger and must form a radical intermediate during the reaction [74], but the half-life of ES• is most likely much shorter than that of Asc•, as ES• will quickly form the disulfide via a combination of two ES• radicals.

## Figures and Tables

**Table 1:** Kinetic parameters of Sec-TrxR and GR/GSH with 2-thioHis disulfide, ESSE, and selenoneine diselenide.

Enzyme	Substrate	*K*_M_ (μM)	*k*_cat_ (min^−1^)	*k*_cat_/*K*_M_ (min^−1^ M^−1^)
Sec-TrxR	2-thioHis disulfide	930 ± 120	4470 ± 300	4.81 × 10^6^
	ESSE	430 ± 105	2900 ± 360	6.74 × 10^6^
	selenoneine	2335 ± 615	6270 ± 790	2.69 × 10^6^
Sec-TrxR/Trx	ESSE	143 ± 6	1925 ± 85	1.34 × 10^7^
GR/GSH	2-thioHis disulfide	2815 ± 500	16,425 ± 1690	5.84 × 10^6^
	ESSE	130 ± 6	20,035 ± 1210	1.53 × 10^8^

**Table 2:** Kinetic parameters of Sec-TrxR with 2-thioHis and EGT oxidized with ^1^O_2_ as substrates.

Enzyme	Substrate	*K*_M_ (μM)	*k*_cat_ (min^−1^)	*k*_cat_/*K*_M_ (min^−1^ M^−1^)
Sec-TrxR	2-thioHis-^1^O_2_	228 ± 36	1306 ± 93	5.73 × 10^6^
	EGT-^1^O_2_	91 ± 15	335 ± 50	3.68 × 10^6^

**Table 3:** Activity of mutant TrxR enzymes and DmTrxR compared to wild-type Sec-TrxR.

Substrate	Enzyme	Normalized Velocity (mol NADPH/min/mol TrxR)	Percent Decrease in Activity Compared to Sec-TrxR (%)
400 μM ESSE	10 nM Sec-TrxR pH 7.0	1430 ± 40	-
	5 nM Sec-TrxR pH 8.0	2670 ± 50	-
	114 nM TrxR-GCCG pH 7.0	23 ± 0.6	98.4
	68 nM TrxR-GCCG pH 8.0	24 ± 0.3	99.1 *
	300 nM TrxR∆3 pH 7.0	2 ± 0.3	99.9
	300 nM TrxR∆3 pH 8.0	1 ± 0.3	99.9 *
	80 nM DmTrxR pH 7.0	87 ± 9	93.9
	80 nM DmTrxR pH 8.0	72 ± 9	97.3 *

* These values are in comparison to Sec-TrxR at pH 8.0, all other values are in comparison to Sec-TrxR at pH 7.0.

## Data Availability

The data are contained within this article and supplemental material.

## Supplementary Materials

The following are available online at https://www.mdpi.com/article/10.3390/antiox11020185/s1, Figure S1: Michaelis–Menten activity plots for the disulfides/diselenides of 2-thioHis, EGT, and selenoneine with Sec-TrxR, Figure S2: Absorbance versus time for GR/GSH, NADPH with 1 mM ESSE, Figure S3: ^1^H-NMR spectra of EGT before and after addition of H_2_O_2_ with structures of EGT and hercynine for reference, Figure S4: MS analysis of 2-thioHis oxidized with ^1^O_2_, Figure S5: RNO bleaching experiments, Figure S6: Absorbance spectra of 2-thioHis with and without rose bengal and light, Figure S7: Michaelis–Menten plots of the reduction in aliquots of 2-thioHis/EGT oxidized by ^1^O_2_ catalyzed by Sec-TrxR, Figure S8: Michaelis–Menten curve for EGT-^1^O_2_ in pH 8.0 ammonium bicarbonate with 200 μM NADPH and 20 nM Sec-TrxR. Figure S9: Control MS experiment showing ^1^O_2_ -oxidized products are stable to ultrafiltration.
