# Supplementary material for: Oxidized Forms of Ergothioneine Are Substrates for Mammalian Thioredoxin Reductase
Source: Antioxidants (Basel). 2022 Jan 19;11(2):185. doi: 10.3390/antiox11020185 (PMC8868364; doi:10.3390/antiox11020185)
Supplement: Supplementary file 1 [file antioxidants-11-00185-s001.zip › antioxidants-1527074-supplementary.pdf]

Supporting Information for Oxidized Forms of Ergothioneine Are Substrates for Mammalian Thioredoxin Reductase†

Kaelyn A. Jenny[1,3], Gracyn Mose[1,3], Daniel J. Haupt[2], and Robert J. Hondal[*1, 2]

[1]Department of Biochemistry, 89 Beaumont Ave, Given Laboratory, Room B413, Burlington, VT 05405

[2]Department of Chemistry, 82 University Place, Innovation Hall, Room E340, Burlington, VT 05405

[3]These authors contributed equally.

†These studies were supported by National Heart, Lung, and Blood Institute grant HL141146 to Robert J. Hondal.

*To whom correspondence should be addressed. Department of Biochemistry, University of Vermont, College of Medicine. 89 Beaumont Ave, Given Laboratory, Room B413, Burlington, VT 05405. Tel: 802-656-8282. FAX: 802-862-8220. E-mail: Robert.Hondal@uvm.edu

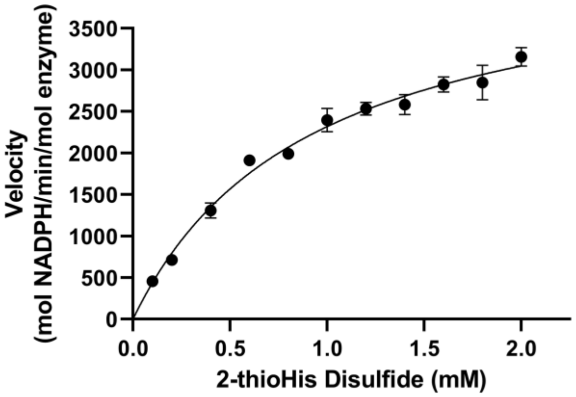

A

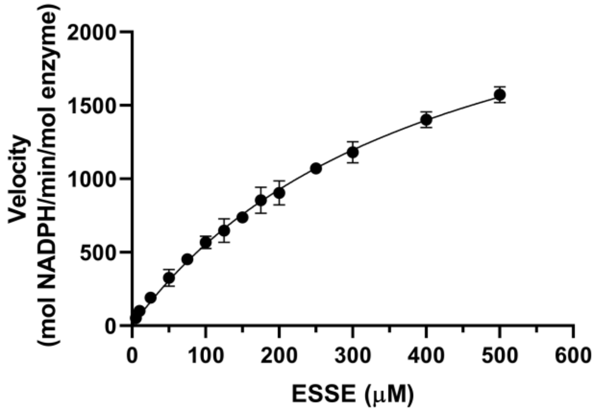

B

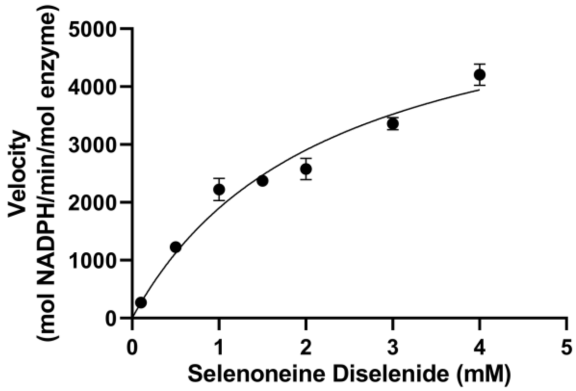

C

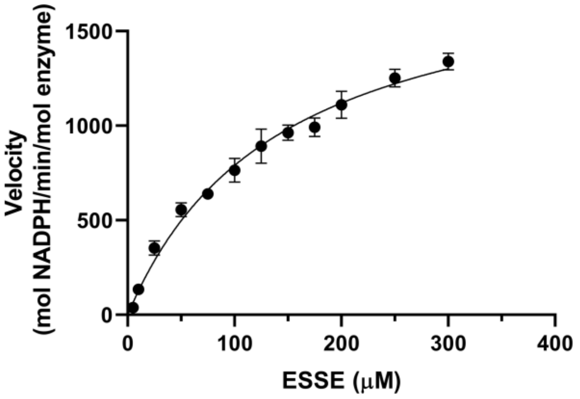

D

Figure S1: Michaelis-Menten activity plots for the disulfides/diselenides of 2-thioHis, EGT, and selenoneine with Sec-TrxR. A) Assay of 2-thioHis oxidized with H_2O_2. B) Assay of EGT oxidized with H_2O_2. C) Assay of 2-thioHis oxidized with H_2O_2 D) Assay of EGT oxidized by H_2O_2 in the presence of 10 μM *E. coli* Trx. The data points in D) represent the corrected data with the activity of 10 μM *E. coli* Trx alone subtracted from the activity in which both ESSE and Trx are present.

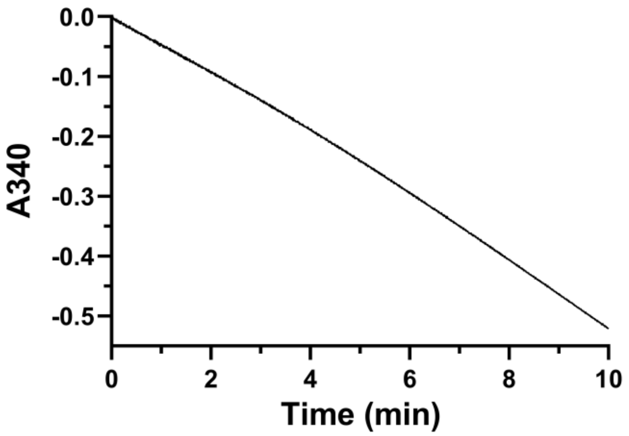

Figure S2: Absorbance versus time for GR/GSH, NADPH with 1 mM ESSE. To a cuvette, 200 μM GSH, 200 μM NADPH, 1 mM ESSE, and 0.60 nM GR were added and the reaction was followed for 10 min. The activity increases with time as substrate is reduced.

NMR experiments with EGT and H_2O_2:To quantify the extent of desulfurization of EGT following treatment with H_2O_2 to produce ESSE we performed quantitative ^{1}H-NMR experiments. The hercynine C2 proton in samples of EGT in D_2O was found to have a long T1 relaxation time of 14.42 seconds. Using this T1 value, quantitative ^{1}H-NMR with 2 dummy scans and 8 scans over ~12 min was performed on two samples: 10 mM EGT dissolved in D_2O and 10 mM EGT with 5 mM H_2O_2 in D_2O (**Figure S3**). A ^{1}H-NMR spectrum was obtained 10 min after adding H_2O_2 to the sample. The hercynine C2 proton at 8.34 ppm is well resolved in ^{1}H-NMR so we used this as the main indicator of desulfurization. With the addition of H_2O_2 to EGT the C2 proton signal increased from 0.02 to 0.03, indicating an increase from 2% to 3% desulfurization upon a 10 min incubation with 5 mM H_2O_2 (**Figure S3**). Several new peaks appeared in the oxidized sample at 3.34, 3.89, 7.15, 7.21, and 7.26 ppm indicating the presence of oxidized species of EGT such as ESSE, hercynine, or other oxidized forms. There was also notable peak broadening and slight peak shifting in the oxidized sample, most evident in the peak at 6.82 ppm (compare to 6.72 ppm peak in unoxidized sample on the left in **Figure S3**) indicating the presence of multiple EGT species of different oxidation states and possibly the

formation and break-down of these species on the NMR time scale. It has been shown that when EGT forms ESSE under aqueous conditions, it can form *S*-monoxide disulfide and *S*-dioxide disulfide, which break down into the sulfenic and sulfinic acids, followed by desulfurization to hercynine, EGT, and sulfurous acid.[9,13] This NMR experiment leads us to conclude that the desulfurization of ESSE producing EGT and hercynine is not extensive in the time-frame of the enzyme kinetics assays.

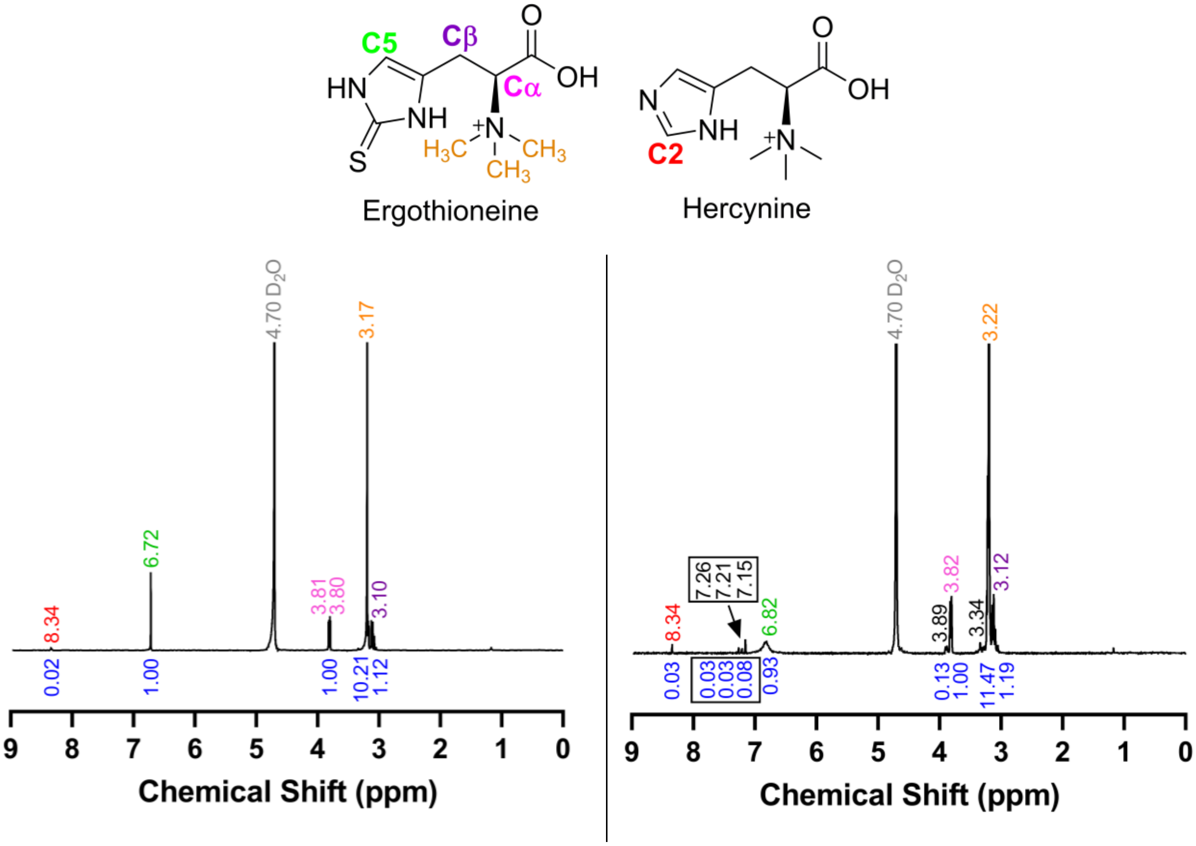

Figure S3: ^{1}H-NMR spectra of EGT before and after addition of H_2O_2 with structures of EGT and hercynine for reference. The carbons of EGT and hercynine are labeled for reference. (left) ^{1}H-NMR spectrum of 10 mM EGT with no H_2O_2. (right) ^{1}H NMR spectrum of 10 mM EGT with 5 mM H_2O_2.

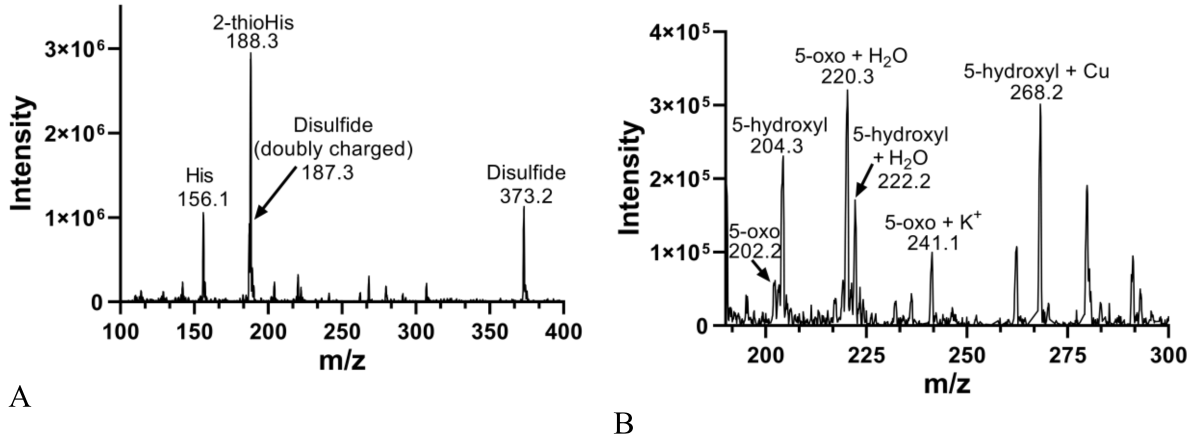

Figure S4: MS analysis of 2-thioHis oxidized with $^1O_2$. A) Full MS spectrum of 2-thioHis following oxidation with $^1O_2$. B) MS spectrum of 2-thioHis oxidized with $^1O_2$ zoomed in to show the 5-oxo product peaks. The main oxidized species of 2-thioHis present was the disulfide form with only small amounts of the 5-oxo and 5-hydroxyl products.

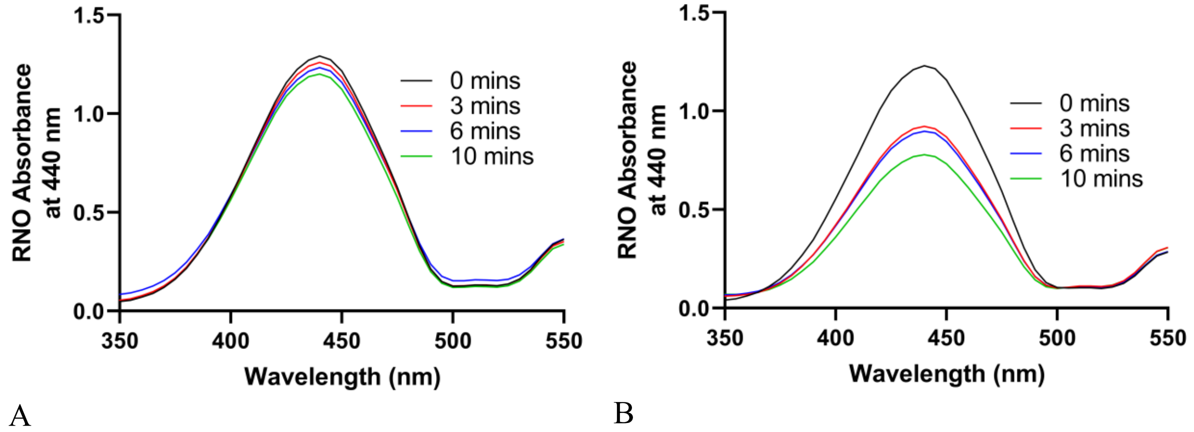

Figure S5: RNO bleaching experiments. A) 10 μM rose bengal with 50 μM RNO in 90% D_2O incubated under light for 20 min. B) 10 μM rose bengal with 50 μM RNO and 8 mM imidazole in 90% D_2O incubated under light for 20 min. The absorbance of RNO at 440 nm decreases dramatically when imidazole, a singlet oxygen scavenger, is added to the reaction.

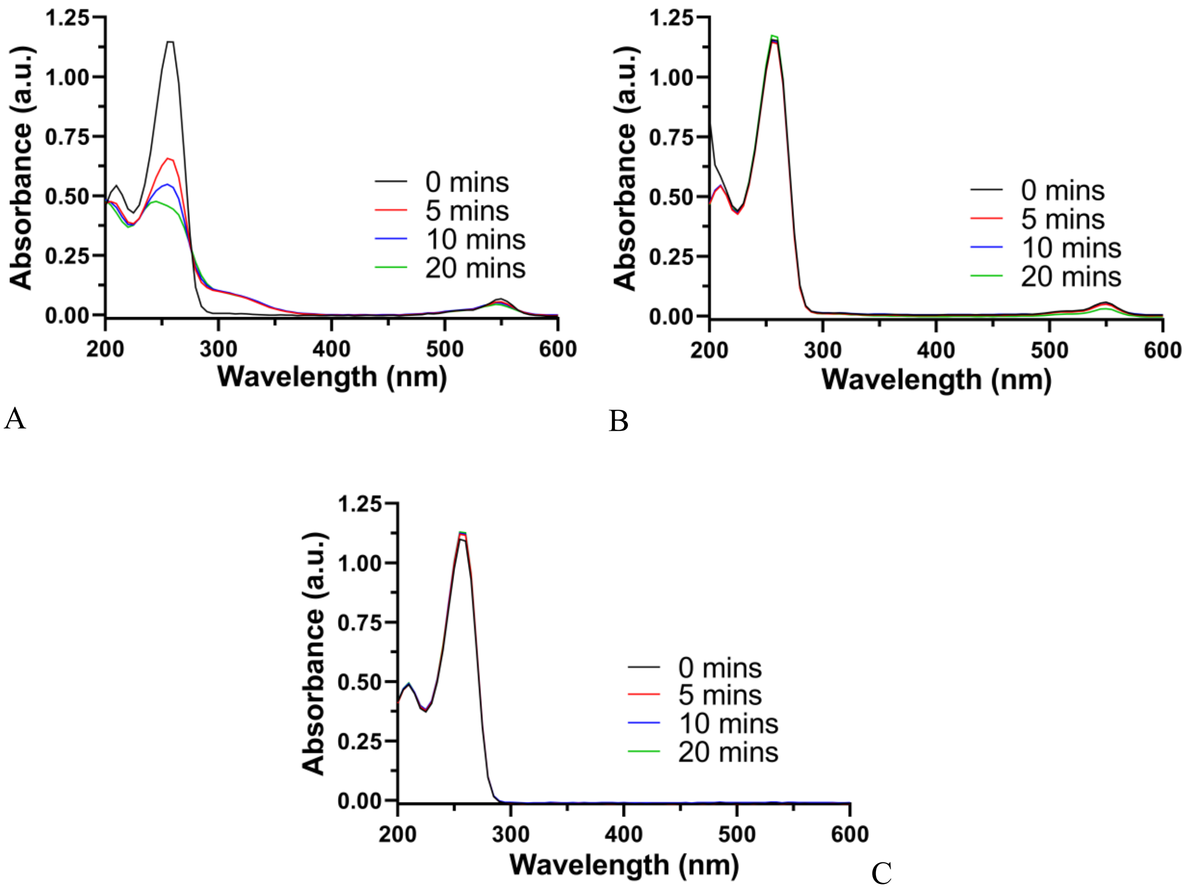

Figure S6: Absorbance spectra of 2-thioHis with and without rose bengal and light. A) 1 mM 2-thioHis and 10 µM rose bengal, irradiated with light for 20 min. B) 1 mM 2-thioHis and 10 µM rose bengal, incubated in the dark at room temperature for 20 min. C) 1 mM 2-thioHis irradiated with light for 20 min. Absorbance of a diluted solution (75 µM 2-thioHis) was measured at 0, 5, 10, and 20 mins.

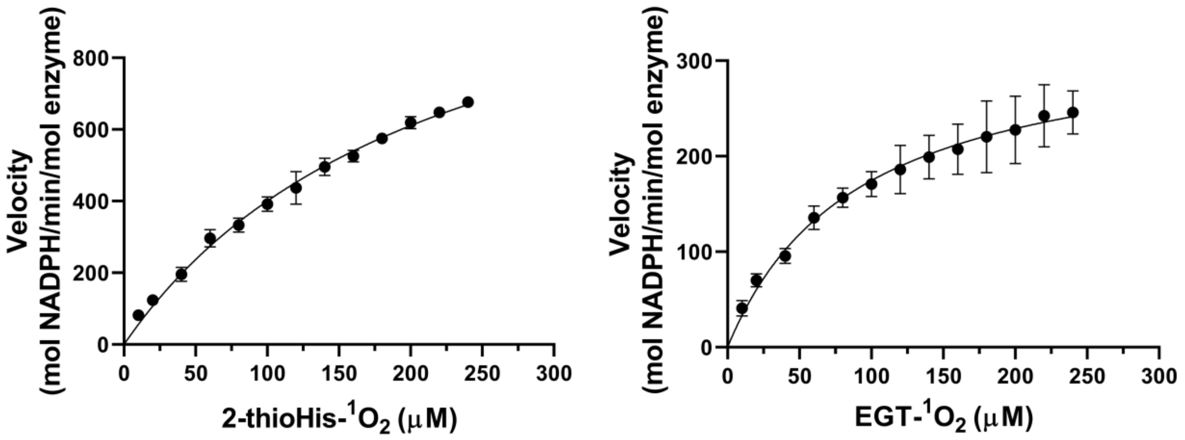

A B

Figure S7: Michaelis-Menten plots of the reduction of aliquots of 2-thioHis/EGT oxidized by $^1O_2$ catalyzed by Sec-TrxR. A) Plot for $^1O_2$ oxidized 2-thioHis and Sec-TrxR. B) Plot for $^1O_2$ oxidized EGT and Sec-TrxR.

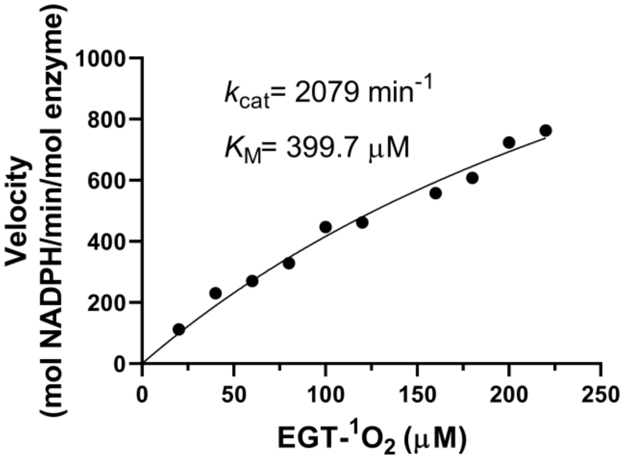

Figure S8: Michaelis-Menten curve for EGT-$^1O_2$ in pH 8.0 ammonium bicarbonate with 200 μM NADPH and 20 nM Sec-TrxR. These are the conditions used for MS analysis of the enzymatic recycling of 5-oxo-EGT. This experiment was performed to ensure that Sec-TrxR had activity in pH 8.0 ammonium bicarbonate buffer prior to MS analysis.

m/z	Identity
198.1	Hercynine
230.1	EGT
244.1	5-oxo-EGT
246.1	5-oxo-EGT and 5-hydroxy-EGT
252.1	EGT + Na
261.1	EGT + EGT + Cu (+2 charge)
268.1	EGT + K and/or 5-hydroxy-EGT+ Na
284.1	5-hydroxy-EGT + K
305.2	EGT + EGT + Cu + CO_2 + CO_2 (+2 charge)
457.2	ESSE
479.2	ESSE + Na
489.2	ESSE S-dioxide (ES(O)$_2$SE)
495.1	ESSE + K

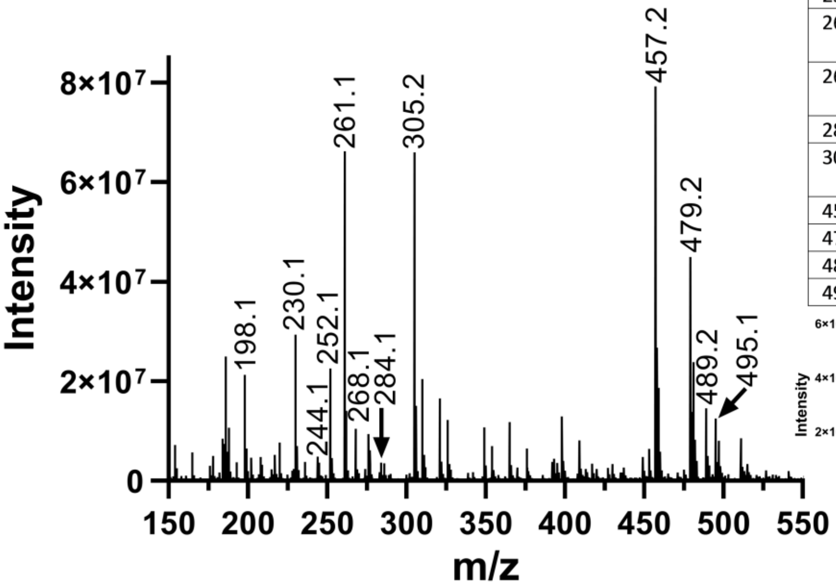

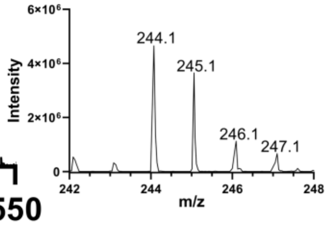

Figure S9: Control MS experiment showing that 5-oxo-EGT, 5-hydroxy-EGT, and ESSE are stable to ultrafiltration. The inset shows a close-up of 5-oxo-EGT and 5-hydroxy-EGT.
